# A Spatial Adaptation Strategy for Safe Campus Open Spaces during the COVID-19 Pandemic: The Case of Korea University

**DOI:** 10.3390/ijerph19159390

**Published:** 2022-07-31

**Authors:** Odilia Renaningtyas Manifesty, Junga Lee

**Affiliations:** 1 Department of Architecture, Korea University, Seoul 02841, Korea; ormreyn@korea.ac.kr; 2 Division of Environmental Science & Ecological Engineering, College of Life Sciences & Biotechnology, Korea University, Seoul 02841, Korea

**Keywords:** open space on campus, spatial resilience, pandemic preparedness, spatial adaptation

## Abstract

Open spaces on campus offer various opportunities for students. However, the coronavirus disease (COVID-19) pandemic has affected students’ comfort when occupying open spaces on campus. The purpose of this study is to investigate possible spatial adaptation strategies for safe campus open spaces during the COVID-19 pandemic. For this research, a case study was conducted using a mixed methodology with behavioral mapping that investigated students’ perceptions at Korea University, Seoul, Korea. A qualitative approach was first conducted with behavioral mapping; the results show that despite some behavioral and spatial changes, people still occupy open spaces on campus for various meaningful activities. A quantitative approach with structural equation modeling (SEM) was also conducted to understand the required spatial modifications to improve the safety of open spaces on campus. The positive correlation between (i) social distancing measures, (ii) health protocols, and (iii) accessibility and occupational comfort with (iv) individuals’ fear of COVID-19 as a positive moderation are the four hypotheses proposed in this study. The results suggest that social distancing measures have no correlation with occupational comfort, while accessibility has the largest positive correlation. Suggestions are presented for providing accessible and equally distributed open spaces on campus to avoid overcrowding. Spatial health protocols are also found to positively correlate with occupational comfort, and the perception of the severity of COVID-19 strengthens this correlation. Tangible physical measures to prevent the spread of the virus are necessary to improve students’ sense of comfort and safety in open spaces on campus.

## 1. Introduction

Open spaces on campus (referred to as OSoC in this paper) serve a large and wide role as a social space, (outdoor) educational space, shared amenity between schools, and connection between the school and the public [1,2,3]. Beyond OSoC’s function as a social setting, research that uses a mixed method of cognitive maps and questionnaires suggests that a well-designed OSoC (e.g., with seating arrangements, vegetation, and thermal comfort) can induce social interactions that lead to an increase in users’ level of happiness and that help shape students’ lives at universities [4,5]. Research by Hanan in 2013 emphasized the current trend in university campuses where students’ learning activities have also shifted to outdoor spaces, resulting in the formation of a strong sense of place and strong attachment to the school [6]. OSoC as one of the cities’ open space suppliers can contribute to various aspects, including urban resilience, when properly designed [7]. To ensure the functionality of OSoC as a social infrastructure in this stressful time due to the pandemic, health and safety measures are urgently needed. This study aims to investigate the current situation of OsoC and the correlation between the spatial character of OsoC that is related to coronavirus disease (COVID-19) prevention and students’ comfort in occupying OsoC. Since a study suggests that people perceive the severity of COVID-19 differently [8], this study also aims to examine the possibility that perceptions of the severity of COVID-19 are a factor that affects students’ perceptions of safe OsoC.

Four hypotheses are proposed in this study.

**H1.**  *Proper social distancing measures (independent variable X1) positively affect the occupational comfort of OsoC during the COVID-19 pandemic (dependent variable Y).*

**H2.**  *Proper spatial health protocols (independent variable X2) positively affect the occupational comfort of OsoC during the COVID-19 pandemic (dependent variable Y).*

**H3.**  *The location and accessibility of OsoC (independent variable X3) positively affect the occupational comfort of OsoC during the COVID-19 pandemic (dependent variable Y).*

**H4.**  *People’s perception of the severity of COVID-19 (moderator variable M) significantly moderates the correlation among social distancing measures (X1), health protocols (X2), accessibility (X3), and the occupational comfort of OsoC (dependent variable Y).*

### 1.1. Urban Resilience in the Pandemic Era

Urban resilience was first studied in relation to climate change. After the UN-Habitat released the Sustainable Development Goals, providing safe and inclusive public spaces has also been characterized as an effort toward city resilience [9]. In a study of public policy and local communities, Shaw refers to urban resilience as a city’s ability to adapt and cope when confronted with sudden change, catastrophic events, or any similar external forces [10]. By this definition, the COVID-19 pandemic can be seen as a major unprecedented phenomenon that challenges cities’ resilience in multiple aspects, especially since cities have been known to be more prone to outbreaks. Developing strategies for resilience should focus on the people and how they perceive the pandemic. People adapt to the pandemic differently because of the unique values and experiences of each individual after a catastrophic event, whether it is a natural or social disaster [11]. A study by Yamazaki in 2021 suggests that people who work from home (telecommuters) appreciate open spaces for stress relief, while non-telecommuters appreciate the human connection and social interaction that often occur in public open spaces [12]. There have been changes in people’s motivations to go to urban green spaces and changes to the perceived meaning of urban green spaces and OSoC specifically during the pandemic [13,14]. Before the pandemic, people needed access to green open spaces for leisure or health benefits [15]. The greatest motivation has now become escaping the strict social distancing measures that occur more often in indoor spaces [16]. In OSoC, although students are more reluctant to engage in social interactions, the number of visitations does not change significantly [17]. In addition to the change in people’s appreciation for public open spaces in cities, the pandemic has significantly affected interpersonal distance. This adjustment of interpersonal distance occurs not only in domestic spaces such as the home but also in other aspects of urban spaces, especially in the hospitality and leisure businesses [18]. Various measures have also been implemented in cities’ social and green infrastructure spaces, such as parks and plazas, to reduce virus transmission between visitors. Ann Forsyth, an urban planning professor at Harvard Graduate School, notes that simply enforcing suppression by limiting people’s movement cannot be implemented in a city forever because it leads to other issues, such as delayed herd immunity and economic fallout [19]. Google Mobility Reports states that throughout 2020, there were significant fluctuations in park visitations in major cities around the world. Although regional restrictions play a large part in these fluctuations, people’s perception of the danger of the COVID-19 virus is also a factor [20]. It is paramount for cities to develop adaptation strategies to contain the impacts of the COVID-19 pandemic and possible future pandemics that focus on the people’s coping mechanisms [21].

In the case of the COVID-19 pandemic, creating more compact and diverse cities, developing apartment typologies that minimize overcrowding, and mitigating the impact on vulnerable communities have been proposed as strategies to reduce the risk of the pandemic [22]. These strategies need to be regularly evaluated because, in most cases, preparedness metrics use only a limited analysis of the correlation, although many variables are involved, and no two regions possess identical needs [21]. As many cities move forward with “living with COVID” scenarios, adaptation strategies for the pandemic need to focus on giving people their lives back without underestimating the transmission risk and the severity of the disease. Four interconnected aspects of overcoming the pandemic must be handled equally to enhance urban resilience: social, spatial, capital, and governance aspects [23]. This study closely observed spatial resilience in public spaces and specifically on OSoC, as one of the core elements that shapes and is shaped by the community. Strategies to achieve spatial resilience include quick and temporary responses or conceptual and longer-term responses. Brooklyn’s Domino Park, with its social distancing circle and grid squares in a plaza in Vicchio, are examples of temporary responses. For longer-term responses, the solutions about preparation for the post-COVID-19 era have been presented in a more utopian manner, as shown in design competitions, such as the Seoul Ideas Competition in 2019 [24]. On university campuses, adaptation strategies usually take the form of short-term solutions, such as social distancing, by implementing new rules, restricting access, and adjusting behavior [25]. However, due to the heavy operational cost, these strategies cannot be used as permanent solutions for spatial resilience against pandemics.

### 1.2. Pandemic Impacts on OSoC

Concern regarding the resilience of public open space during the pandemic is required not only at the city level but also at the community or institutional level, such as university campuses. A study by Szczepańska and Pietrzyka noted that university students are negatively affected by the strict lockdown policy and its domino effect of remote learning, which prevents them from having face-to-face interaction with their peers [8]. Limited access to public open spaces further adds to the deterioration of their physical and psychological well-being during this pandemic. Social distancing policies often lead to a lack of social interaction among all university members, but students are more vulnerable to stress due to social isolation [26]. Furthermore, the closures of the OSoC affect not only university members but also the surrounding community, as they decrease the supply of open spaces in cities, which could lead to the unequal distribution of open spaces [27]. A model by Brigham and Women’s Hospital showed that failure to implement a proper mitigation plan on campuses could put 16% of faculty members at risk of getting the infection per semester, and this number could increase to 15 times higher for students [28]. Various dimensions need to be considered when making mitigation plans to reopen campuses to minimize the risk of the plans being unsuccessful or only partially successful [29]. The first dimension concerns policies and must be synergized with local governments. As their main strategy in battling the spread of the virus, most countries have implemented “test, trace, and isolation” protocols with different degrees of application [30]. The second dimension concerns the individual and social behavior of university members. Preventive behavior, such as wearing a mask, maintaining proper social distance, and routine testing, can prevent up to 96% of infections on university campuses [31]. The last dimension concerns the spatial aspects of campuses that not only further prevent the spread of the virus but can also influence preventive behaviors. Following the general anti-virus guidelines that state indoor spaces pose a higher risk of virus transmission than outdoor open spaces, Korea University in South Korea has implemented some learning space modifications as pandemic responses, such as widening the gap between seats in classrooms, putting a transparent barrier in cafeterias, and limiting entrance points to make tracing systems easier.

To create resilience in campus areas in the pandemic era, spatial adaptation should be implemented not only in classrooms but also in other areas, such as public open spaces. Similar to public open spaces that play an important role as a social infrastructure where people socialize [32], OSoC also plays a significant role for students in providing a space to have meaningful activities and as an instrument to engage the campus with its surroundings [33]. However, OSoC has a major difference from general open spaces due to its users being mainly students who have distinctive needs compared to the general public. Before the pandemic started, open spaces on the Korea University Seoul campus had always been crowded with students performing various activities related to their campus life. OSoC of Korea University was a place for students to experience various leisure, extracurricular, and academic activities year-round. Since the pandemic started, preventive measures have been implemented with a focus on indoor spaces, although it is important to also modify outdoor spaces to ensure their usefulness for university campuses. To understand the strategies that are needed to achieve spatial adaptation in OSoC and to identify proper variables for this study, a review of various articles on approaches to creating safe OSoC and public spaces, in general, was conducted. Only measures that can be implemented in OSoC are included in the review as shown in Table 1. The strategies for spatial adaptation as a response to the pandemic can be divided into three categories. The first category addresses social distancing measures that can be afforded by the space, such as vegetation placement that prevents the formation of large crowds. The next category addresses health protocols and includes providing instruments on site to help prevent virus transmission, such as hand sanitizers. The last category involves the accessibility and distribution of the space.

## 2. Methodology

**Figure 1:**
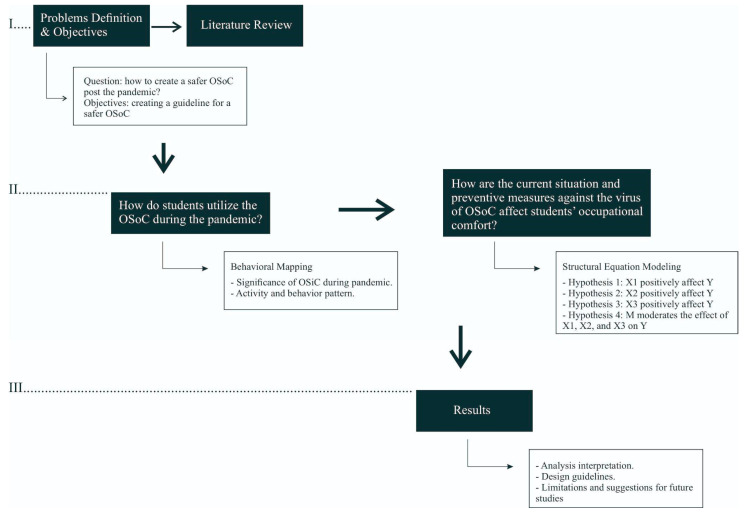
The research framework.

Figure 1 shows the framework of this study in which three main steps are included. The first step includes defining the issue this paper attempts to solve and relevant literature review as presented in chapter one. The second step includes organizing variables and hypotheses based on the literature review, and data gathering and analysis. Direct observation and questionnaires were used to gather the data, while data analysis was conducted with behavioral mapping to investigate OSoC occupation during the pandemic and structural equation modeling (SEM) to determine which aspects need to be improved to make it a safer space by testing the proposed hypotheses. The final step includes the presentation of the results and the analytical interpretation of it that was then used to construct a set of design guidelines. This step also includes the limitations of this research that should be addressed in future studies.

### 2.1. Sample and Site Location

**Figure 2:**
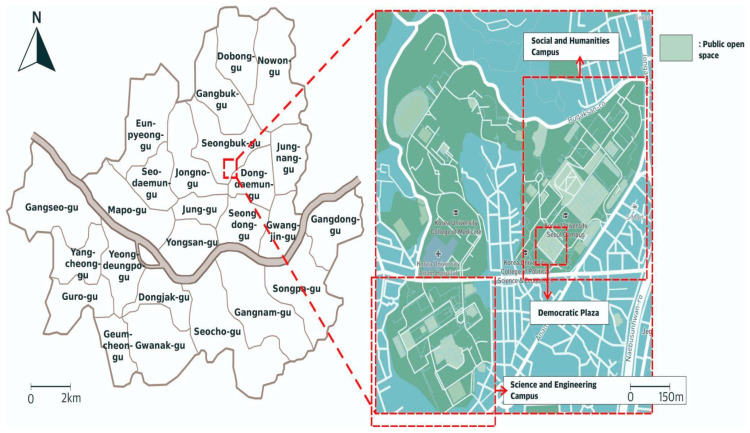
Map of the site (Korea University) and its location in Seoul Metropolitan City.

This study was conducted qualitatively and quantitatively, to provide more comprehensive results. A qualitative approach was conducted with behavioral mapping in one of the main plazas of Korea University to investigate how OSoC is utilized during the pandemic. The quantitative study was conducted with data collected from questionnaires distributed at Korea University Seoul Campus (referred to as Korea University in this study) to students at the graduate level. Korea University was chosen as the focus of the study for several reasons. First, the location in Seoul Metropolitan City makes Korea University very suitable for this study, which focuses on OSoC as part of cities’ green and social infrastructure [33]. Second, as seen in Figure 2, Korea University has a large number of open spaces in the forms of public plazas, inner courts, and pedestrian ways that are well distributed in its large area. OSoC in this paper is limited to areas that allow people to perform stationary activities (sitting, smoking, talking with others, etc.) and excludes spaces used mainly for movement, such as alleys. Finally, Korea University has implemented an open campus concept so that anyone can freely walk around the campus. The campus is also a location for two subway stations and several bus stops. These features are important because, in this paper, location and accessibility are the two main factors of the occupational comfort of the OSoC. Democratic Plaza was chosen as the specific site for behavioral mapping because it is not only located near a subway station but also relatively accessible to both the Social and Science Campus students.

The respondents were limited to graduate school students for two main reasons. First, graduate school students have come to school more often than undergraduate students during the pandemic, which makes them more familiar with the OSoC situation during the pandemic. The obligation to conduct research forces graduate students to regularly visit the campus despite the online class policy that has been imposed since early 2020. Second, they utilize campus facilities more often than undergraduate students for research purposes. This has made their movement around the campus and access to OSoC more frequent. The respondents were further limited to students from only 2 main campuses, namely, the Science and Engineering campus and the Social and Humanities campus. The medical school at the northern Korea University that houses the university’s hospital was excluded due to its access limitations for nonpatients and nonfaculty members. The questionnaires were distributed during a two-week period between 2 and 16 December 2021. Out of 98 responses obtained from the online questionnaire, 6 responses were classified as “straightliners” because the same answers were selected for all questions. These responses were deemed invalid.

### 2.2. Behavioral Mapping

This study used space-centered behavioral mapping to observe the behavioral patterns occurring on the site. Behavioral mapping is a powerful tool to understand humans’ relationship with their surroundings, especially in the built environment, and has been widely used in studies on time geography, post occupancy evaluation, and public open spaces [42]. The observation was conducted four times in four different time periods in November 2022 to gain wide data coverage. The first and second observations were conducted during lunch time (2 p.m.) and after-work time (6 p.m.), respectively, on a weekday. The third and fourth observations were conducted at the same time but on a weekend. Each observation was conducted for a 30-min period, and the observation was conducted directly by the author. November was chosen because it is in the middle of the semester, and the weather is considered acceptable for various outdoor activities, with a daily average temperature of 11 °C and humidity of 59%. The results of the observations were recorded on a map that contained the following information: types of activities; gender of visitors; number of visitors; and major people movements.

### 2.3. Path Model and Survey Data Analysis

**Figure 3:**
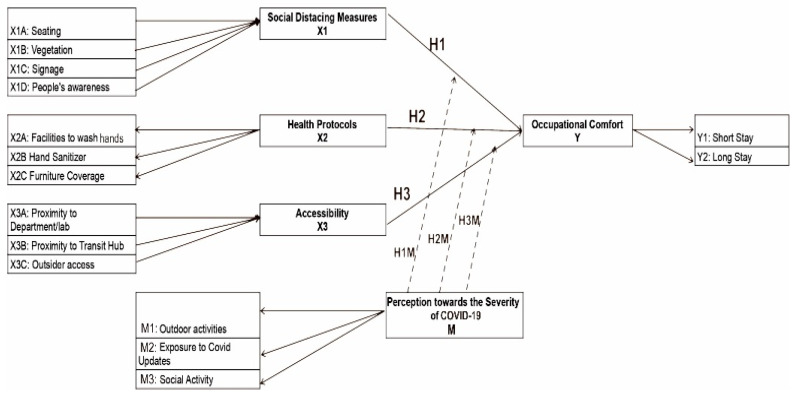
The statistical path model for the study.

The questionnaire used in this study followed a path model that consisted of 5 latent variables (or constructs) derived from the literature review. As seen in Figure 3, the constructs were divided into three independent variables, one moderator variable, and one dependent variable. The independent variables were the following spatial features of the OSoC related to COVID-19 prevention measures: (i) social distancing measures; (ii) health protocols; (iii) accessibility of the OSoC. Social distancing measures were defined as spatial features that force visitors to practice social distancing. Indicators of this construct included (i) how well the existing spatial arrangements, such as seating and vegetation layout, reinforced the social distancing measures, (ii) the presence of signage asking visitors to maintain a safe distance from each other, and (iii) visitors’ awareness of following the rules. Health protocols were any other preventive measures in addition to social distancing that could help reduce virus transmission. Indicators of this variable included hand sanitizing facilities and furniture protection or coverage. The last independent variable was the accessibility of the OSoC and was defined as how the location of the OSoC could contribute to preventing virus spread. Its indicators included the proximity of the OSoC to the participants’ respective departments and external access recognition. The dependent variable was the occupational comfort of the OSoC and was defined as users’ willingness and comfort level in performing activities in the OSoC during the pandemic. Users’ perception of the severity of the pandemic was the moderator variable that was suspected to play a part in affecting the correlation between the spatial features of the OSoC and occupational comfort. It was measured by the following three indicators: the tendency to have outdoor activities after the pandemic; the intensity of social interactions; the daily exposure to COVID-19 updates. The last variable was occupational comfort, which was measured by how comfortable the students felt in staying for a short and long time in the OSoC during the pandemic.

The path model was then translated into a quantitative questionnaire in which the respondents agreed or disagreed on a 5-point Likert scale with 15 statements that represented the 15 indicators. Figure 3 shows that the measurement model included formative (arrows toward the construct) and reflective indicators (arrows toward the indicator) that depend on the causal relationship with each latent variable. The determination of the type of indicator was based on the author’s judgment and initial plan of the logical relationship between the indicators and the construct [43]. A total of 15 indicators distributed among the 5 constructs were included in the model. Since variables X1 and X3 were measured by the existing situation and condition, these indicators cause a constituent concept toward the constructs. The variables X2 and M were measured by conditions that reflect their constructs, making them reflective indicators. The data from the questionnaire were analyzed by using a partial least squares structural equation model (PLS-SEM) with SmartPLS software, which is well known to be a reliable tool to analyze path models. SEM was used rather than a conventional multiple regression because SEM has the ability to present more significant statistical relationships [44]. Furthermore, the PLS-SEM method was chosen for this study because it is suitable for quantitative exploratory research, which is the nature of this study, and because the relatively small sample size is not a limitation.

The data analysis was conducted through 2 main steps that began with inputting the data to SmartPLS 3.0 for a process called path modeling and analysis. After all responses from the questionnaires were inputted, a path model that follows Figure 2 was built. Afterward, a measurement model was conducted as the first step to evaluate how well the indicators predicted the construct. For the reflective indicators, validity and reliability tests were conducted by measuring the outer loading and average variance extracted (AVE) for the validity test and by measuring Cronbach’s alpha to test the reliability. These measurements were performed by running the PLS algorithm function on the software. The threshold of the AVE and Cronbach’s alpha to be considered valid and reliable was 0.5 and 0.6, respectively [45]. For the formative indicators, the reliability test was not applicable because there was no correlation between the indicators [46]. Testing the validity of formative indicators is more complex and requires a few more steps. First, each indicator’s collinearity had to be checked through the variance inflation factor (VIF); the value should be lower than 3.3. Next, the significance of each indicator should be measured with t statistics. If the result says the indicator is significant to measure the construct (minimum value of 1.96), then the indicator should be retained. However, if the t statistic is considered insignificant, then an outer loading analysis should be conducted; if the value is at least 0.5, then the indicator should not be deleted [47]. Both the VIF and t statistics measurements were performed by running the bootstrapping function under the ‘Calculate’ menu. The second step of the analysis was the structural model, which evaluated the correlation among the independent, moderator, and dependent variables to verify the proposed hypotheses. This step included the measurement of the path coefficients by running the PLS algorithm function again to determine the positive or negative correlation between the independent and dependent variables. Next, the t statistic and P value were measured with a bootstrapping process to determine the significance of each correlation. The measurement of the structural model was also applied to the moderator variable to determine its moderating effect.

## 3. Results

### 3.1. Activity and Behavioral Pattern

**Figure 4:**
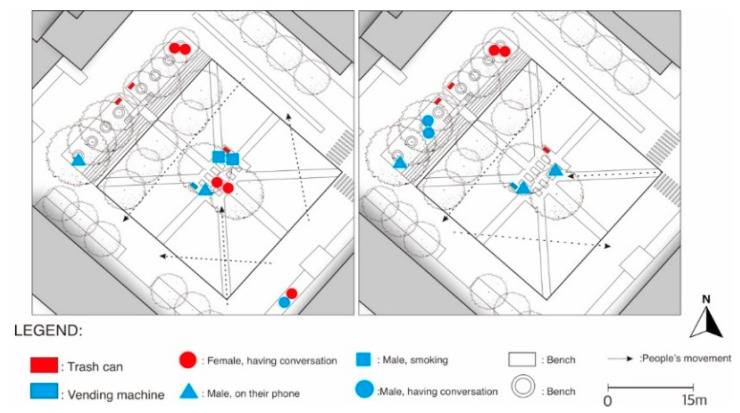
Behavioral map of people engaged in various activities during a weekday at Democratic Plaza (on the left: at 6 p.m., on the right: at 2 p.m.).

Figure 4 shows that people gathered and performed stationary activities in two main locations: under the tree row and near a vending machine and a trash can that is equipped with an ashtray. People occupied the seating under the tree canopy from the outer side first, indicating that they wanted to maintain a safe distance from each other. The place also seemed to be more meaningful, with more people spotted on the site in the evening time as a place for people to relax before going home from work. Activities such as smoking and talking with acquaintances were also seen to occur at a later time. The mobility pattern shows that the plaza is quite accessible from various directions and that the designated paths within the plaza are used only to go to the seating located at the center of the plaza but not to cross the plaza. Due to the conducive weather during the observation time, stationary activities were seen more than people’s movement.

**Figure 5:**
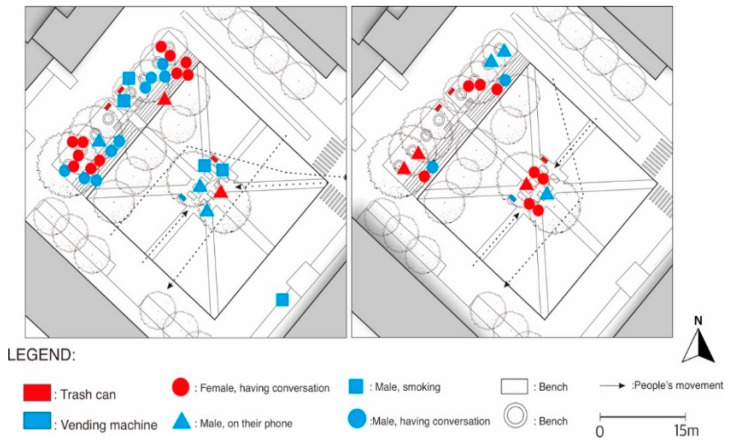
Behavioral map of people engaged in various activities during a weekend at Democratic Plaza (on the left: at 6 p.m., on the right: at 2 p.m.).

According to Figure 5, more people were seen occupying the plaza, especially during the evening. People were gathered at the same spot as the weekday observation, with an obvious increase in the number of people sitting under the tree row on the northwest part of the plaza. There is no indication of a decrease in the number of visitors compared to the pre-COVID-19 time. Slight behavioral change was identified where some people chose to sit scattered on the steps instead of on the unoccupied benches under the trees. This indicates that people tried to create a safe distance between each other. The age difference between the visitors varied more, indicating that the plaza is accessible to visitors who are nonstudents or even nonfaculty members. There was no difference in the mobility pattern between weekdays and weekends. Overall, direct observation of the site suggests that despite the pandemic, the Democratic Plaza still plays an important role for students and some people from external communities, although people’s behavior showed that they are more vigilant about the risk of COVID-19.

### 3.2. Measurement Model Evaluation

Variables X1 and X3 were first analyzed together because both have formative indicators. The result of indicator collinearity measurement showed that no indicator in either construct was at a critical level, enabling a significance analysis process through the bootstrapping method. However, after the significance check, indicator X1A of the “Social Distancing Measure” construct and indicator X3B of the “Accessibility” construct had t values lower than 1.96. After the outer loading analysis, indicators X1A and X3B continued to appear to be invalid; hence, they had to be removed from the model. Table 2 shows the complete measurement model evaluation; all indicators besides X1A and X3B are considered valid. The rest of the variables (X2, M, and Y) were evaluated next, and all indicators were deemed to be valid and reliable. The result is shown in Table 3, with all AVE values higher than 0.5, which is the threshold for valid convergence.

### 3.3. Structural Model Evaluation

**Figure 6:**
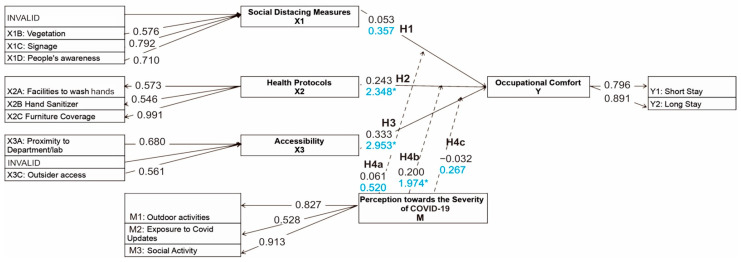
PLS algorithm and bootstrapping result of the proposed model. * *p* < 0.05.

After the evaluation of the measurement models was performed, the next step was to evaluate the structural model to test the hypotheses. Before testing the hypotheses, a predictive relevance evaluation, also known as the Stone-Geisser’s Q^2^ value, was conducted to check the model’s suitability [48]. The Q^2^ value can be obtained by running the blindfolding process. The proposed model had a Q^2^ value of 0.163, which indicates that the model was well constructed. Next, the proposed model’s path coefficients and t statistic values were examined to prove the hypothesis. Figure 6 shows the values of both path coefficients (written on top) and t statistics (written below the path coefficient) for each independent and dependent variable. For the moderator variable, only the significance value matters to check the hypothesis. The final result of the measurement model shows that hypothesis 1 is not supported, hypotheses 2 and 3 are supported, and hypothesis 4 is only partially supported. Even though there is a positive correlation between social distancing measures and occupational comfort, it is not significant. For health protocols and accessibility, the correlations are positive and significant. Furthermore, the perception of the severity of COVID-19 is only significant to strengthen (positive path coefficient and t statistics >1.96) the correlation between health protocols and occupational comfort. It is, however, not found to be significant as a moderator for the correlation between both social distancing measures and accessibility to occupational comfort.

## 4. Discussion

Looking at the significance of OSoC for university students, this study assumed that during the COVID-19 pandemic, students at the Korea University campus would be more comfortable occupying the OSoC if it had spatial characteristics that did not contribute to the spread of the virus. This study also assumed that the positive correlation between the spatial characteristics of OSoC and occupational comfort is stronger when students perceive COVID-19 as a serious threat to their health. The study is meaningful in that several aspects of an OSoC were individually analyzed based on their respective theory and used together to construct a model to analyze which spatial characteristics contribute to the occupational comfort of the OSoC. This study also considered the possibility of individuals’ perception of the severity of COVID-19 as a moderating effect in pushing the need to create a more pandemic-resilient OSoC. Based on the literature review of the meanings and importance of OSoC for university students, the pandemic impacts, and its possible spatial adaptations, a research model was constructed. Although the behavioral mapping analysis confirmed that there was no significant change in the number of OSoC visitors during the pandemic, several behavioral changes were identified between post- and pre-COVID-19, such as avoiding sitting too close to other visitors. This finding supports an earlier study by Alnusairat et al. that shows that there are more students who consider increasing their frequency in visiting the OSoC than students who would stop their visits post-COVID-19 although there is also a tendency to reduce social interaction [17]. Several prior studies suggest that a higher temperature and extremely low or high humidity pose a higher risk of virus transmission [37,38]. The vibrancy of Democratic Plaza might indicate that students are more comfortable visiting OSoC when the temperature is lower with moderate humidity similar to the climatic attributes of the plaza during the observation time.

Three main spatial aspects were examined in this study that are considered to contribute to the creation of safer OSoC in the pandemic era. However, the first aspect that was observed, the social distancing measure, was not statistically proven to positively affect the occupational comfort of the OSoC. Although further research needs to be conducted to empirically understand the reason for this, previous studies have suggested that some people have a tendency to see open spaces as safer than indoor spaces and that they were perceived as stress relief during the pandemic [12,13,16]. This implies that strict social distancing measures might not be seen as mandatory in outside spaces as in indoor spaces. In addition, accessibility has a significant and positive correlation with occupational comfort. This result supports a previous study that suggests that equal access to open spaces reduces travel times and therefore reduces the risk of infections [41,43]. However, accessible but not equally distributed open spaces can be dangerous and can increase the risk of transmission due to overcrowding [39,40]. This is similar to the accessibility concept in the compact city mentioned in Table 1; it can be beneficial, but without proper mitigation, disadvantages might arise due to high density. Unlike social distancing measures, which are noticeable by the practices and behavior of people, health protocols, such as the installation of hand washing facilities, are more tangible. This is probably why the health protocols variable was found to positively affect the perceived comfort of OSoC, while the social distancing measure was not found to affect it. This finding is further supported by the result of the moderator variable, which is significant only to strengthen the correlation between health protocols and occupational comfort. Health protocols are tangible measures that students can see being implemented, and they provide a stronger sense of safety when the perception of the severity of COVID-19 is high [49]. Given this phenomenon, providing tangible and physical measures to prevent the spread of the virus in OSoC is necessary.

## 5. Conclusions

Our study emphasizes the importance of OSoC and the urgent need to create safe OSoC in the time of a global pandemic. Although studies related to the impacts of the COVID-19 pandemic on urban resilience continue to increase, there are still very limited sources that examine approaches to spatial adaptation in educational institutions. This study combines two approaches to understand the value of OSoC in the pandemic era and to develop adaptation strategies to create safer open spaces in campus areas during the COVID-19 pandemic. The behavioral mapping used in this study suggests that with the current minimum alterations to prevent the spread of the virus, people still occupy the OSoC for various meaningful activities throughout the week. This finding led us to consider the importance of spatial adaptation for more pandemic-resilient OSoC so that the risk of virus transmission can be minimized. To discover the needed spatial adaptations, SEM was used to analyze the interrelationships between various spatial characteristics and occupational comfort. Based on the analytical results, we suggest strategies for pandemic-resilient OSoC as follows.

First, providing tangible measures is necessary to raise the occupational comfort of OSoC. Compared to environmental design that forces the implementation of social distancing, the existence of preventive instruments and technologies such as sanitizers and antiviral surfaces makes visitors feel safer in OSoC. This suggestion is further supported by the significance of individuals’ fear of the virus in moderating the correlation between the two abovementioned variables. The results indicate that when individuals possess a greater fear of contracting the virus, they are more comfortable performing activities in OSoC after seeing preventive instruments at the site. Second, students are more comfortable visiting OSoC that is near to their place so that travel times can be minimized. This study suggests that compared to social distancing measures and other health related protocols, quick access is more favorable and results in better occupational comfort. Future campus development should address this suggestion by ensuring equal distributions of OSoC in university areas. With equal distribution, OSoC can contribute not only to the well-being of students but also to the surrounding communities by ensuring open space supply in cities. Although combining quantitative and qualitative approaches is the strength of this study, some limitations need to be addressed. First, the methodology cannot provide empirical reasons for the correlations. Future research should include additional methods, such as interviews or forum group discussions, to provide more thorough results. Second, more environmental attributes of OSoC need to be addressed in the future to create more comprehensive guidelines.

## Figures and Tables

**Table 1:** Summary of the strategies for creating spatial adaptation in OSoC and Public Open Spaces during the pandemic.

Category	Approach
Social distancing measures	Preventing large gatherings can be achieved actively through signage and passively through design intervention [34].
	Passive prevention through street furniture or street art is preferable due to its effectiveness, pleasantness and non-hostile nature [34].
Other health protocols	The usage of materials that are easy to clean that cover surfaces with protective materials and the maintenance of the humidity level help minimize the spread of the COVID-19 virus through design [35].
	Easy access to facilities where people can wash their hands or access hand sanitizer can minimize the spread of the virus [34].
Accessibility	Access should be restricted for patients, close contacts, and people with high risk such as public transportation users who are not first sanitized [36].
	Creating a compact city where citizens do not have to commute far to have access to urban amenities lowers virus transmission [35]. However, some negative effects are also associated with living in a compact city during this pandemic, such as an increase in depression symptoms and overcrowding [37,38].
	The distribution of open spaces is more important than creating large spaces during the pandemic. Ensuring equal and easy access to open spaces is pivotal when considering the benefits of open spaces in the pandemic era [39,40].
Perception of the severity of COVID-19	Although people’s awareness of COVID-19 does not stop them from going to parks, it does change their behavior and attitude while visiting parks [41].
	To prevent panic over the virus within communities, knowledge of the correct facts and preventive measures is necessary [28].
	Students tend to occupy OSoC despite unfavorable conditions such as bad weather [17], and this seems to also be applicable to the pandemic situation based on the current conditions at Korea University.

**Table 2:** Measurement Model Evaluation: Formative Indicators.

Indicators	VIF	T Statistic	Outer Loading
X1B: Vegetation The vegetation layout of the OSoC helps disperse large gatherings.	1.401	2.267 *	0.576
X1C: Signage There is signage that reminds people to maintain a safe distance.	1.401	1.050 *	0.792
X1D: People Awareness People are seen practicing social distancing measures.	1.032	2.249 *	0.71
X3A: Proximity to Department There is an OSoC near my department or lab.	1.06	2.448 *	0.68
X3C: Outsider Access Non-faculty members can freely access KU’s OSoC.	1.149	2.118 *	0.561

* *p* < 0.05.

**Table 3:** Measurement Model Evaluation: Reflective Indicators.

Indicators	Outer Loading	AVE	Cronbach’s Alpha
X2A: Access to Hand Wash There is a facility to wash hands near the OSoC.	0.573	0.506	0.666
X2B: Access to Hand Sanitizer There is a hand sanitizer facility near the OSoC.	0.546		
X2C: Furniture Coverage The furniture at OSoC is covered with material that is easy to clean/disinfect.	0.991		
M1: Outdoor Activities I limit going outside to reduce the risk of getting COVID.	0.827	0.523	0.693
M2: Exposure to COVID News I update myself daily on the latest case of COVID-19.	0.528		
M3: Social Activities I limit my social activities to reduce the risk of getting COVID.	0.913		
Y1: Short Time Stay I feel comfortable staying at the OSoC for a short time (less than 30 min).	0.796	0.714	0.606
Y2: Long Time Stay I feel comfortable staying at the OSoC for a long time (more than 30 min).	0.891		

## Data Availability

The datasets used and analyzed in this study are available from the authors upon a reasonable request.
